# Identification of a Simplest Hypervalent Hydrogen Fluoride Anion in Solid Argon

**DOI:** 10.1038/s41598-017-02687-z

**Published:** 2017-06-07

**Authors:** Meng-Chen Liu, Hui-Fen Chen, Chih-Hao Chin, Tzu-Ping Huang, Yu-Jung Chen, Yu-Jong Wu

**Affiliations:** 1 National Synchrotron Radiation Research Center, 101 Hsin-Ann Road, Hsinchu Science Park, Hsinchu 30076 Taiwan; 2 Department of Medicinal and Applied Chemistry, Kaohsiung Medical University, 100, Shih-Chuan 1st Road, Kaohsiung, 80708 Taiwan; 3 Department of Physics, National Central University, Jhongli City, Taoyuan County 32054 Taiwan; 4 Department of Applied Chemistry, National Chiao Tung University, 1001, Ta-Hsueh Road, Hsinchu, 30010 Taiwan

## Abstract

Hypervalent molecules are one of the exceptions to the octet rule. Bonding in most hypervalent molecules is well rationalized by the Rundle–Pimentel model (three-center four-electron bond), and high ionic bonding between the ligands and the central atom is essential for stabilizing hypervalent molecules. Here, we produced one of the simplest hypervalent anions, HF^−^, which is known to deviate from the Rundle–Pimentel model, and identified its ro-vibrational features. High-level *ab inito* calculations reveal that its bond dissociation energy is comparable to that of dihalides, as supported by secondary photolysis experiments with irradiation at various wavelengths. The charge distribution analysis suggested that the F atom of HF^−^ is negative and hypervalent and the bonding is more covalent than ionic.

## Introduction

The octet rule indicates that atoms of the main group elements tend to gain or lose electrons in order to have eight electrons in their valence shells^1, 2^, similar to the electronic configuration of the noble gases. Therefore, to attain this fully filled electronic configuration, atoms combine to form molecules by sharing their valence electrons to form chemical bonds. This rule is especially applicable to the period 2 and 3 elements. Most molecules are formed by following this rule and the bonding structure of molecules can be easily recognized by using Lewis electron dot diagrams. This concept is well established in many general chemistry textbooks, along with three exceptions to the octet rule: (1) radicals (such as NO), (2) electron-deficient species (such as B_2_H_6_), and (3) hypervalent species (such as SF_6_).

Although, Lewis and Langmuir debated the nature of hypervalent chemical bonding as early as the 1920s^3, 4^, the term “hypervalency” was first defined by Musher in 1969 as a molecule with a central atom of group 15–18 in any oxidation state other than the lowest oxidation state^5^. To date, the bonding nature and classification of hypervalent molecules have been debated. Pauling first used sp^3^d hybridization to tentatively explain the hypervalency of PCl_5_ and SF_6_^6^. Later, Rundle and Pimentel proposed the three-center four-electron (3c-4e) bonding model (also known as the Rundle–Pimentel model) to rationalize bonding in hypervalent molecules without the need for expanded octets^7–10^. The main picture of the 3c-4e bond is similar to the model of two-center one-electron (2c-1e) bonds proposed earlier by Sugden^11^, and the 3c-4e bond can be thought of as the bonding of a hypervalent molecule consisting of two collinear 2c-1e bonds, with the remaining two nonbonding electrons localized on the ligands.

In the 1990s, a series of theoretical calculations performed with the Hartree-Fock level indicated that using d-orbital hybridization (sp^3^d^2^) to explain bonding in hypervalent species was unnecessary^12, 13^. Subsequently, theoretical analysis of the electron localization function (ELF) for hypervalent species bonded with electronegative ligands, such as SF_6_ and PCl_5_, indicated that the ligands can pull electron density away from the central atom, and therefore, the central atom has fewer than eight valence electrons^14, 15^. Thus, this finding supported the term “hypercoordination”^16, 17^ to describe such species. In addition, the ELF analysis of some hypervalent species with weakly electronegative ligands, such as P(CH_3_)_5_, As(CH_3_)_5_, and Te(CH_3_)_6_, showed that the population of valence electrons on the central atoms was greater than eight.

Meanwhile, the concepts of recoupled pair bonding^18^ and charge-shift bonding^19, 20^ were developed to explain the stability of hypervalent species. Very recently, an alternative definition of hypervalency based on the analysis of atomic charge maps was proposed^21^. A new parameter called the valence electron equivalent, γ, was introduced to describe the effective number of valence electrons on any particular atom. This new definition really echoes ELF calculations^14, 15^ for those hypervalent species with strong ionic bonding characters defined by Musher’s definition^5^ are reclassified as hypercoordinate. However, using this definition, some species previously generally accepted as obeying the octet rule, such as O_3_ and N_2_O, would be considered as hypervalent.

## Results and Discussion

**Figure 1:**
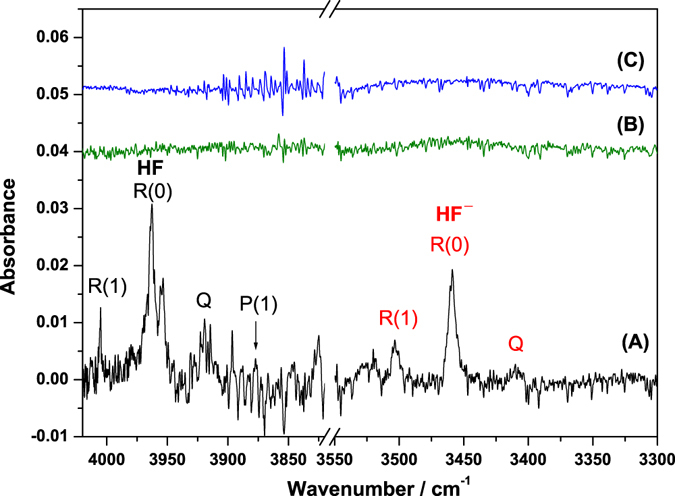
Partial IR spectra of electron-bombarded (2000 eV, 0.3 mA) matrix samples at 10 K. (**A**) CH_3_F/Ar (1/500), (**B**) CH_4_/Ar (1/500), and (**C**) Ar. The ro-vibrational transitions of the observed bands are assigned.

Despite some criticism of the term and concept of hypervalency, hypervalent species of the second-row elements^22–24^ and hypervalent all-hydrido species^25, 26^ have rarely been observed experimentally. We produced hydrogen fluoride anions in an argon matrix by electron bombardment of excess Ar containing a small proportion of methyl fluoride during matrix deposition. By optimizing the experimental conditions, clear triplet features was observed at 3503.4, 3458.8, and 3409.4 cm^−1^. This triplet pattern and peak intervals are similar to the ro-vibrational structure of neutral HF isolated in solid Ar^27, 28^, as shown in Fig. 1(A). Figure 1(B) and (C) depict partial infrared (IR) spectra of electron-bombarded CH_4_/Ar and pure Ar samples under the same experimental conditions. The comparison of these spectra clearly shows that the triplet feature near 3458.8 cm^−1^ is associated with the H−F stretch. Moreover, as only simple molecules can rotate in rare-gas matrices, the observation of similar rotational parameters indicates that the carrier might be an ionic counterpart of HF. However, the identification of the H−F stretch of HF^+^ at 3090.5 cm^−1^ via photoelectron spectroscopy^29^ excludes the possibility of the new IR feature being associated with HF^+^.

**Figure 2:**
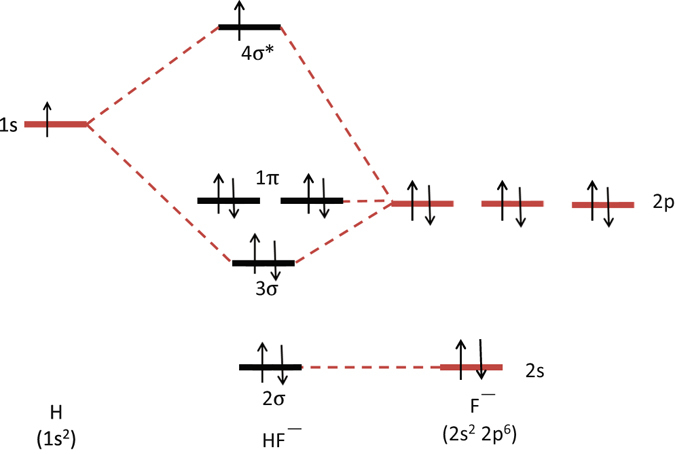
Qualitative molecular orbital diagram of HF^−^. The 2σ orbital, coming from the F 2 s orbital, is non-bonding. The 3σ orbital is a combination of the F 2p_z_ and H 1 s orbitals and is bonding, whereas the F 2p_x_ and 2p_y_ orbitals cannot interact with the H 1 s orbital due to different symmetries and serve as non-bonding orbitals. The 4σ* antibonding orbital is the counterpart of the 3σ bonding orbital. From this diagram, the bond order of HF^−^ is calculated to be 0.5.

**Figure 3:**
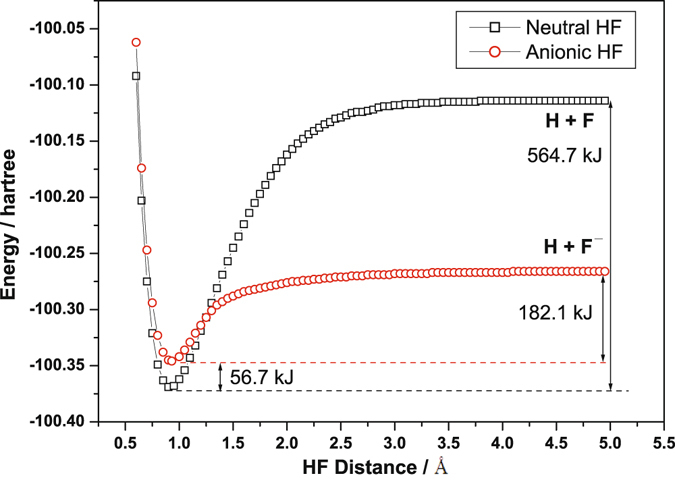
Potential energy curves of neutral and anionic HF in their ground states, calculated at a CCSD(T)/Aug-cc-pVQZ level of theory. The energy difference between the two species is listed with zero-point energy correction. In comparison, the experimental bond dissociation energy (D_0_) of HF is reported to be 565.3 kJ mol^−1^ ^40^.

Although the early theoretical work predicted that the ground state of HF^−^ (*X* ^2^Σ^+^) was entirely repulsive^30^, recent high-level calculations showed the existence of a bound state for this species^31, 32^. The qualitative molecular orbital diagram, as depicted in Fig. 2, also shows the two-center three-electron (2c-3e) σ half-bonding character of HF^−^ (*X* ^2^Σ^+^). The 2c-3e hemibonds have in common with electron rich (3c-4e) hypervalent to share a common physics though, both of them belong to the class of Charge Shift bonds^33–36^. We therefore used the coupled cluster method including triple excitation (CCSD(T))^37, 38^ with a basis set of Aug-cc-pVQZ^39^ to construct the potential energy surface of HF^−^ (*X* ^2^Σ^+^) with the variation of the distance between H and F atoms, along with that for HF (*X* ^1^Σ^+^) for comparison, as depicted in Fig. 3. The calculated equilibrium distance of HF is 0.918 Å, which is almost identical to the experimental value (0.917 Å)^40^, whereas the equilibrium distance of HF^−^ (0.935 Å) is slightly longer than that of HF. Although the adiabatic electron affinity of HF is calculated to be −56.7 kJ mol^−1^, the dissociation energy of HF^−^ to H + F^−^ is predicted to be about 182.1 kJ mol^−1^, which is comparable to the bond strength of a typical halogen–halogen bond^41^. The vibrational frequency of the HF stretch of HF^−^ predicted by various theoretical methods is listed in Table 1, along with that of HF for comparison. The *ab inito* calculations, including CCSD(T)^37, 38^, QCISD(T)^37^, and MP2^42^, give a similar predicted bond length and vibrational wavenumber for neutral and anionic HF, whereas the predicted frequency of the HF stretch deviates by ~160 cm^−1^ from the experimental value. In contrast, the density-functional method, B3LYP^43, 44^, gives a closer prediction. We used scaling factors of 0.960 for the *ab inito* methods and 0.974 for the density-functional method, obtained from the ratio between the theoretical and experimental results, to yield a scaled vibrational frequency for HF^−^ in the region of 3542–3387 cm^−1^. Thus, our experimental observation of new lines near 3459 cm^−1^ is in good agreement with this region, supporting the assignment of the new lines to HF^−^. Moreover, using the rotational constant predicted at a CCSD(T)/aug-cc-pVTZ level of theory to simulate the ro-vibrational structures of HF^−^ also showing a good agreement with our experimental observation, as compared in Fig. S1 in Supplementary Information (SI).

**Table 1:** Comparison of the predicted bond distance, harmonic vibrational wavenumbers, IR intensities, and rotational constants of HF, HF^−^, and DF^−^ with experimental values.

	CCSD(T)	QCISD(T)	MP2	B3LYP	Ar matrix^a^
**HF**					
r/Å	0.921	0.921	0.922	0.924	
ν/cm^−1^	4125	4120	4126	4070	3918.8
Int / km mol^−1^			121	111	
B_e_/cm^−1^	20.8	20.7	20.7	20.6	20.9^b^
**HF** ^**−**^					
r/Å	0.940	0.940	0.940	0.948	
ν/cm^−1^	3690	3682	3683	3479	3409.4
Int/km mol^−1^			502	349	
B_e_/cm^−1^	20.1	19.9	19.9	19.6	22.3
**DF** ^**−**^					
ν/cm^−1^	2675	2674	2673	2522	2526.6
Int/km mol^−1^			231	156	
Ratio^c^	0.7250	0.7262	0.7258	0.7250	0.7411
B_e_/cm^−1^	10.5	10.5	10.5	10.3	15.9

The basis set used for all calculations is Aug-cc-pVTZ.

^a^Position of the Q band.

^b^The rotational constant of gaseous HF is 20.9557 cm^−1^ ^53^.

^c^Defined as the ratio between the wavenumbers corresponding to the D-isotopic species and HF^−^.

For further confirmation of the spectral assignments of HF^−^, electron bombardment of a mixture of CD_3_F/Ar (1/500) was performed. Experimental procedures similar to those for CH_3_F/Ar were followed, and a representative IR spectrum in the range 2400–2700 cm^−1^ is shown in Fig. S2. In this system, the triplet band was found at 2592.1, 2560.4, and 2526.6 cm^−1^. The obtained deuterium isotopic shift ratio was comparable to the theoretical values, as summarized in Table 1.

**Figure 4:**
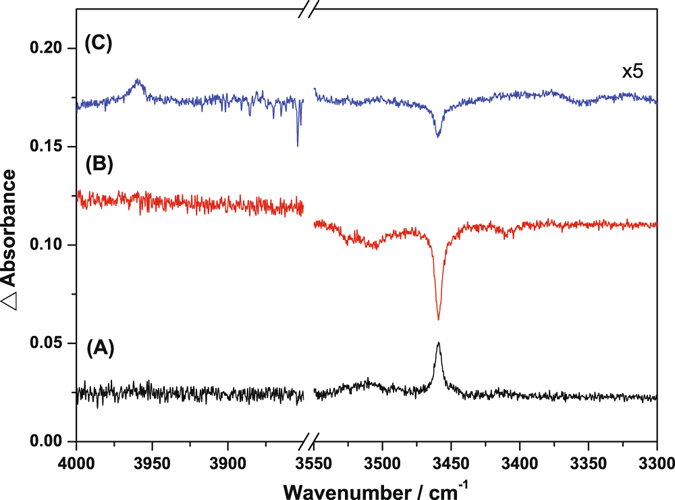
Difference IR spectra of the electron-bombarded matrix samples at 10 K upon secondary photolysis with (**A**) 210 nm and (**B**) 385 nm, and (**C**) maintained in the dark for 4 h.

In addition, secondary photolysis of the matrix sample at 210 nm increased the intensity of the HF^−^ bands, but photolysis at 385 nm decreased the intensity of these bands. The difference IR spectra, as shown in Fig. 4, were obtained by subtraction of the spectrum recorded before from that recorded after irradiation or storage in the dark. Lines pointing upward indicate the production of species, whereas those pointing downward indicate the destruction of species. Maintaining the matrix sample in darkness for 4 h also resulted in a decay of the HF^−^ band intensity, but formation of its neutral counterpart (HF) was observed. This observation is consistent with the exothermic reaction for detachment of an electron from HF^−^. However, photolysis of HF^−^ at 210 and 385 nm did not result in the formation or destruction of HF. Moreover, irradiation at 210 nm for a prolonged time also depleted HF^−^. As the bond dissociation energy of HF^−^ is predicted to be 182.1 kJ mol^−1^ (~657 nm), we thus performed irradiations at 450, 525, and 675 nm and the depletion of HF^−^ was observed upon irradiation with these three wavelengths. However, photolysis with 675 nm increased the band intensity of HF. The photon energy of 675 nm is insufficient to dissociate HF^−^, but this might detach electron away from this anion to form its neutral counterparts; the representative difference spectra were available in Fig. S3. Moreover, the photodissociation of HF^−^ took place at these shorter wavelengths might be because there are corresponding excited states in these photon energy region. The preliminary calculations of the first six low-lying excited states of HF^−^, as summarized in Table S1, support this possibility. In addition, irradiation of the matrix sample with 210 nm for a short period resulted in the formation of HF^−^, that can also be accounted through this calculation of the excited states. The excitation with 210 nm matches less the electronic states of this anion, whereas enhance the migrations of H and F^−^ around the matrix to combine with each other^45^. Further UV-visible spectral measurements of this anion are in the process.

To understand the formation mechanism of HF^−^, we studied the dependence of the formation of this anion on the emitting electron energy and electron flux, as depicted in Fig. S4. The formation of HF^−^ does not have a linear dependence on the electron bombardment energy and electron flux, indicating that this species was formed indirectly. Moreover, the absence of observable HF^−^ formation at a low bombardment energy (200 eV) also suggested that considerable energy is required for electron bombardment to cause complete fragmentation of methyl fluoride. Taken together, we concluded the formation of HF^−^ in the current study is via combination of H atom with F^−^ anion, but not from association of electrons and neutral HF.

## Conclusion

Finally, HF^−^ was produced by electron bombardment of the Ar matrix containing a small amount of CH_3_F during deposition, and its ro-vibrational bands corresponding to the HF stretching mode were assigned and compared with those obtained using high-level calculations. The theoretical vibrational frequencies agree well with the experimental values, but the rotational constants do not. These deviations might be related to the property of chemical bond in hypervalent molecules affected by matrix environments. Further theoretical works on the HF^−^ anion sitting in an Ar lattice will performed for better understanding of this hypervalent molecule. Irradiation of the matrix sample at 385, 450, and 525 nm resulted in HF^−^ bond dissociation, but irradiation at 675 nm resulted in the electron detachment of this anion, which agreed well with the predicted bond dissociation energy of 182.1 kJ mol^−1^ (~657 nm). In contrast, irradiation at 210 nm increased amounts of HF^−^ owing to increased mobility and combination of H atoms and F^−^ anions in the matrix. The band intensity of HF^−^ was monitored as a function of the energy of electron bombardment and electron flux, and thus HF^−^ was suggested to form by association of H and F^−^ atoms in the solid matrix. The charge distribution of HF^−^ calculated by the atoms in molecules (AIM) method^46^ showed that the fluorine and hydrogen atoms have valence electron equivalent (γ) of 9e and 1.5e, respectively. Hence, the F atom in HF^−^ is hypervalent according to Durrant’s definition, and the bonding character is more covalent than ionic. This study is the first demonstration on the experimental observation of the one of simplest heteroatom hypervalent anion and might assist our further understanding of hypervalent molecules consisted of the first and second row elements.

## Experimental and Theoretical Methods

The experimental setup has been described previously^47, 48^. IR absorption spectra covering the spectral range of 450–5000 cm^−1^ were recorded with an interferometric spectrometer (Bruker v80) equipped with a KBr beam splitter and a Hg–Cd–Te detector cooled to 77 K. Typically, 400 scans at a resolution of 0.25 cm^−1^ were recorded at each stage of an experiment.

The anions were produced by electron bombardment of a gaseous sample containing a small proportion of CH_3_F during the deposition of an Ar matrix. An electron beam at 200–3000 eV with a current of 100−500 μA was generated with an electron gun (Kimball Physics, Model EFG-7). Typically, a gaseous mixture of CH_3_F/Ar (1:500) was deposited over a period of 4 h with a flow rate of 5–8 mmol h^−1^. Experiments with CH_4_/Ar (1:500), CD_3_F/Ar (1:500), and Ar were conducted using the same conditions. Photoirradiation experiments were performed with synchrotron radiation at BL03 of NSRRC (~5 mW at 210 nm), and a light-emitting diode (bandwidth ~10 nm, 350 mW at 385 and 450, 170 mW at 525 and 675 nm). Ar (99.9999%, Scott Specialty Gases), CH_3_F (99.5%, Matheson), and CD_3_F (deuterium ~99%, Aldrich) were used without further purification, except for a freeze–pump–thaw procedure at 77 K.

The energies, equilibrium structures, vibrational wavenumbers, and IR intensities of the species were calculated using the Gaussian 09 program^49^. The geometries of HF and HF^−^ were fully optimized at CCSD(T), QCISD(T), MP2, and B3LYP levels of theories with a basis set Aug-cc-pVTZ and CCSD(T)/aug-cc-pVQZ were used for obtaining more accurate relative energy for the electron affinity and bond dissociation energy of this species. The vertical excitation energies and oscillator strengths of the first six excited states of HF^−^ were calculated with equation-of-motion coupled cluster (EOMCCSD)^50^ and time dependent (TD−B3LYP)^51^ methods with a basis set Aug-cc-pV5Z. The charge distribution of the species was calculated with the AIMALL^46^ and the ro-vibrational structures were simulated by a PGOPHER program^18, 29, 45, 52^.

## Electronic supplementary material

Supplementary information
