# Supplementary material for: Identification of a Simplest Hypervalent Hydrogen Fluoride Anion in Solid Argon
Source: Sci Rep. 2017 Jun 7;7:2984. doi: 10.1038/s41598-017-02687-z (PMC5462790; doi:10.1038/s41598-017-02687-z)
Supplement: Supplementary file 1 — Supplementary information [file 41598_2017_2687_MOESM1_ESM.doc]

**Supplementary Information**

Identification of a Simplest Hypervalent Hydrogen Fluoride Anion in Solid Argon

Meng-Chen Liu,^1^ Hui-Fen Chen,^2^ Chih-Hao Chin,^1^ Tzu-Ping Huang,^1^ Yu-Jung Chen,^3^ and Yu-Jong Wu^1,4^,*

^1^National Synchrotron Radiation Research Center, 101 Hsin-Ann Road, Hsinchu Science Park, Hsinchu 30076, Taiwan

^2^Department of Medicinal and Applied Chemistry, Kaohsiung Medical University, 100, Shih-Chuan 1st Road, Kaohsiung 80708, Taiwan

^3^Department of Physics, National Central University, Jhongli City, Taoyuan County 32054, Taiwan

^4^Department of Applied Chemistry, National Chiao Tung University, 1001, Ta-Hsueh Road, Hsinchu 30010, Taiwan

*E-mail: yjw@nsrrc.org.tw

**Table S1** Vertical excitation energies (nm) and oscillator strengths (*f*, listed in parentheses) of HF^−^ predicted by EOM-CCSD and B3LYP with basis sets of Aug-cc-pVQZ and Aug-cc-pV5Z.

|  | EOM-CCSD | |  | B3LYP | |
| --- | --- | --- | --- | --- | --- |
| State | Aug-cc-pVQZ | Aug-cc-pV5Z |  | Aug-cc-pVQZ | Aug-cc-pV5Z |
| *1* ^2^Σ | 531  (0.2216) | 604  (0.2046) |  | 544  (0.2065) | 628  (0.1216) |
| *2* ^2^Σ | 321  (0.2170) | 464  (0.2392) |  | 359  (0.2158) | 519  (0.3217) |
| *3* ^2^Π | 318  (0.4494) | 392  (0.4301) |  | 331  (0.4059) | 408  (0.4226) |
| *3* ^2^Π | 318  (0.4494) | 392  (0.4301) |  | 331  (0.4059) | 408  (0.4226) |
| *4* ^2^Π | 233  (0.0156) | 264  (0.0037) |  | 256  (0.0504) | 296  (0.0137) |
| *5* ^2^Π | 233  (0.0156) | 104  (0.0000) |  | 256  (0.0504) | 276  (0.0404) |

**Figure S1** Comparison of the simulated spectra of HF^－^ by PGOPHER program^1^ with the experimental observation. The rotational constant = 20.1 cm^－1^ was used for the vibrational ground and upper states. The black solid line depicted the spectrum simulated at 10 K and red dash line for 20 K. The experimental spectrum was recorded after irradiation of the electron bombarded matrix sample with 210 nm at 10 K. The Q-branch of HF^－^ in the solid Ar was induced; the similar result was reported for HF in solid Ar.

^1^ PGOPHER, A Program for Simulating Rotational, Vibrational and Electronic Spectra, C. M. Western, University of Bristol, http://pgopher.chm.bris.ac.uk

**
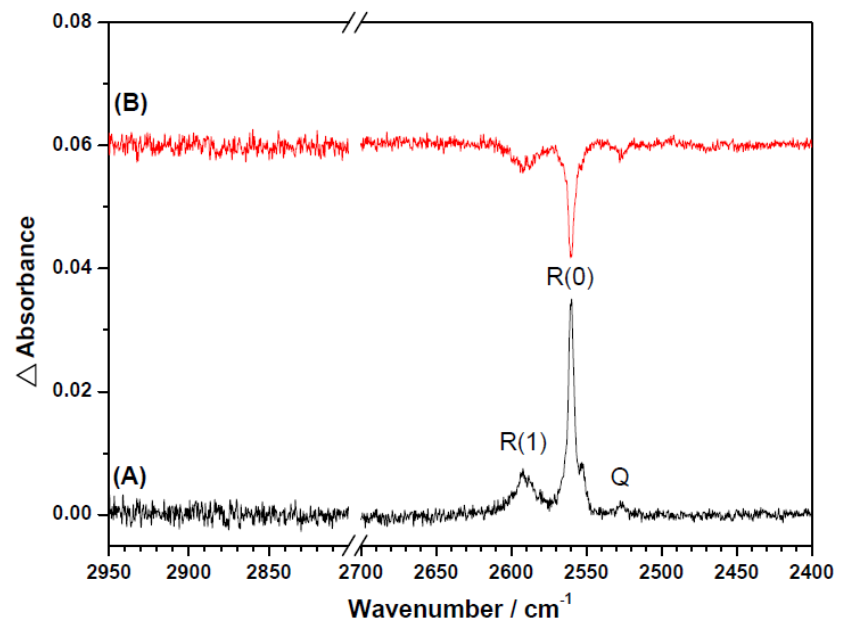
**

**Figure S2** Difference IR spectra of the electron bombarded matrix sample CD_3_F/Ar (1/500) upon irradiation with (A) 210 nm and (B) 385 nm. The representative bands are associated to ro-vibrational DF stretching mode of DF^−^ and assignments are made for each band. The observation of the absence of the absorption band of DF reported near 2895.4 cm^−1^ is consistent with the result of irradiation of HF^−^.

**Figure S3** Difference IR spectra of the electron bombarded matrix sample CH_3_F/Ar (1/500) upon irradiation with (A) 525 nm and (B) 675 nm. Assignments of bands are made. The increase of the intensity of methyl fluoride is due to photoinduced recombination of the nearby fragments upon electron bombardment. The increase of HF was only observed upon irradiation with 675 nm and might because the photon energy of 675 nm is insufficient to dissociate HF^－^, but leads the electron to leave away the anion.

**Figure S4** The integrated band intensity of the HF stretching mode of HF^−^ as a function of (A) energy of electron bombardment with a fixed current of 0.3 mA and (B) electron current with fixed electron energy of 2000 eV.
